# Eastern India Collaboration on Multisystem Inflammatory Syndrome in Children (EICOMISC): A Multicenter Observational Study of 134 Cases

**DOI:** 10.3389/fped.2022.834039

**Published:** 2022-03-11

**Authors:** Snehamayee Nayak, Prakash Chandra Panda, Basudev Biswal, Sunil Kumar Agarwalla, Amit Kumar Satapathy, Pradeep Kumar Jena, Krishna Mohan Gulla, Debasmita Rath, Anuspandana Mahapatra, Pravakar Mishra, Debashree Priyadarshini, Samarendra Mahapatro, Saurav Nayak, Rashmi Ranjan Das, EICOMISC Study Group, Majhi Subash Chandra Kumar

**Affiliations:** ^1^SCB Medical College, Sardar Vallabhbhai Patel Post Graduate Institute of Paediatrics (SVPPGIP), Cuttack, India; ^2^Department of Pediatrics, Veer Surendra Sai Institute of Medical Sciences and Research (VIMSAR), Sambalpur, India; ^3^Department of Pediatrics, Institute of Medical Sciences (IMS) and SUM Hospital, Bhubaneswar, India; ^4^Department of Pediatrics, Maharaja Krushna Chandra Gajapati (MKCG) Medical College and Hospital, Berhampur, India; ^5^Department of Pediatrics and Biochemistry, All India Institute of Medical Sciences (AIIMS), Bhubaneswar, India

**Keywords:** SARS-CoV-2, MIS-C, PIMS-TS, COVID-19, coronary arterial lesions, Kawasaki disease (syndrome), developing country, low and middle income country (LMIC)

## Abstract

**Background:**

Few single center studies from resource-poor settings have reported about the epidemiology, clinical feature and outcome of multisystem inflammatory syndrome in children (MIS-C). However, larger data from multi-center studies on the same is lacking including from Indian setting.

**Methods:**

This retrospective collaborative study constituted of data collected on MIS-C from five tertiary care teaching hospitals from Eastern India. Children ≤ 15 years of age with MIS-C as per the WHO criteria were included. Primary outcome was mortality.

**Results:**

A total of 134 MIS-C cases were included (median age, 84 months; males constituted 66.7%). Fever was a universal finding. Rash was present in 40%, and conjunctivitis in 71% cases. Gastro-intestinal and respiratory symptoms were observed in 50.7% and 39.6% cases, respectively. Co-morbidity was present in 23.9% cases. Shock at admission was noted in 35%, and 27.38% required mechanical ventilation. Fifteen (11.2%) children died. The coronary abnormalities got normalized during follow-up in all except in one child. Initial choice of immunomodulation had no effect on the outcomes. Presence of underlying co-morbidity, lymphopenia, thrombocytosis, hyponatremia, increased LDH (>300 U/L), and hypoalbuminemia were the factors significantly associated an increased mortality.

**Conclusions:**

MIS-C has myriad of manifestations. Underlying co-morbidity, lymphopenia, thrombocytosis, hyponatremia, increased LDH (>300 U/L), and hypoalbuminemia were associated with an increased mortality. No difference in outcome was noted with either steroid or IVIg or both. Coronary artery abnormalities resolved in nearly all cases.

## Introduction

The Severe Acute Respiratory Syndrome Corona Virus-2 (SARS-CoV2) is a global pandemic caused by a Novel Corona Virus Disease (COVID-19) affecting all age groups from neonates to older adults (1–4). The clinical manifestations vary widely ranging from asymptomatic or mild disease (majority of cases) to serious life-threatening conditions. Since the onset of the pandemic, COVID-19 has caused mostly asymptomatic or minor infections in children with an overall incidence of 1–5%, though this figure might be underestimated (5).

During the initial part of the COVID-19 pandemic, several cases of multisystem inflammatory syndrome (MIS) were reported from Europe and North America (6–8). Subsequently, various agencies (World Health Organization [WHO], center for Disease Control [CDC], and Royal College of Pediatrics and Child Health [RCPCH]) circulated case definitions for MIS temporally associated with COVID-19 in children and adolescents (MIS-C) or Pediatric Multisystem inflammatory syndrome temporally associated with SARS-CoV-2 (PIMS-TS) (9–11). Of all these case definitions, WHO MIS-C definition is mostly preferred as it is precise while including most of the cases (12). MIS-C or PIMS-TS is an unusual post-infectious hyper-inflammatory condition mimicking Kawasaki Disease (KD), but with a greater degree of cytokine storm, severity, and poorer outcome. It usually occurs 4–6 weeks following the SARS-CoV2 infection. The clinical spectrum ranges from mild disease with persistent fever to KD like illness or severe life-threatening condition with shock and MODS (multi-organ dysfunction) leading to death (12–14). In the largest series of MIS-C including 518 children from USA, the mortality rate was 2%, which is very less compared to the disease severity (15). A similar figure has been shown by various systematic reviews (12, 14). Another important yet peculiar complication is the development of coronary artery abnormalities (CAAs) that have been shown to develop in 8 to 24% of the cases (16).

Few single center studies from resource-poor settings have reported about the epidemiology, clinical feature and outcome of MIS-C. However, there is a scarcity of large multi-center data on the same including that from Indian setting (13, 17, 18). Moreover, as COVID-19 has become endemic in most parts of the world, MIS-C cases will occur sporadically posing challenges in the management. So, adequate information regarding this condition is need of the hour, and the answer is supposed to come from large multi-center studies.

## Objectives

To describe the following in children with MIS

- Clinical course, risk factors for severe disease, and treatment outcomes.
- Demographic factors, clinical presentations and laboratory findings.

The primary outcome was mortality, and secondary outcomes were demographic & clinical presentations, clinical course, risk factors for severe disease, laboratory findings, and treatment outcomes in children with MIS-C.

## Materials and Methods

This retrospective collaborative study constituted of data collected on MIS-C from five tertiary care teaching hospitals (Government and Private) from Eastern India, over a period of 13 months (October 2020 to October 2021). The study included children of ≤ 15 years of age who had MIS-C as per the WHO criteria (9). Children with incomplete data were excluded from the study. Ethical committee of all five study sites approved the study from ethical perspective (waiver was obtained due to retrospective nature of the collected data).

Before initiation, principal investigators of all five sites met to discuss the standard operating procedure (SOP) for data collection. Data were collected in a pre-designed data extraction form developed by WHO for reporting of MIS-C (19). The data included information about demography, clinical profile, laboratory parameters including inflammatory markers, treatment (including details of Steroid, IVIG, or both or none, any other immune-modulator, antimicrobials, supportive care), and outcome.

### Laboratory Assessment

RT-PCR or cartridge-based nucleic acid amplification test (CBNAAT) were used for detection of SARS-CoV-2 from secretions of the upper respiratory tract as per Indian Council of Medical Research (ICMR) guidelines (20). In children with negative reports, SARS-CoV-2 IgG assay was employed. The SARS-CoV-2 IgG assay is a chemiluminescent microparticle immunoassay (CMIA, Abbott Core Laboratory, IL) intended for the qualitative detection of IgG antibodies to SARS-CoV-2 (against the spike receptor binding domain) in human serum. Laboratory abnormalities were defined as per our previously published collaborative study on the epidemiological profile of SARS-CoV-2 (3). Echocardiography was done to measure ejection fraction, and to look for coronary dilation/aneurysm) at baseline, at 2 weeks, and at 4–6 weeks. The coronary artery abnormalities (CAAs) were reported as “z-scores” (21). A general approach to the cases at each of the five participating sites is shown in Supplementary Figure 1.

### Treatment Categories and Outcome Assessment

Case management was based on the American College of Rheumatology (ACR) clinical guidance for MIS-C (version 1 and 2) (22, 23). Generally, in mild cases no anti-inflammatory drug was administered and patient was evaluated with all blood investigations along with ECG and echocardiography. In severe cases (life threatening conditions like shock, hypotension, and congestive cardiac failure), IV pulse Methyl prednisolone (MP) was given in a dose of 10–30 mg/kg/day for 3–5 days followed by oral steroids (2 mg/kg/day) tapered over 2–3 weeks. IVIg was administered initially in those having features of Kawasaki disease (KD) at a dose of 2 g/kg. Some children required both MP and IVIg. In all severe cases, IV Ceftriaxone and Doxycycline or Azithromycin were started empirically (Scrub typhus is endemic in this part of India, and can have a similar presentation). Antibiotics were stopped after cultures were negative. Oxygen, fluid restriction, cardiac bed, inotropes, and other supportive measures were administered depending on the need. After discharge, children were followed up after 2 weeks in the outpatient department. In children with CAAs, follow-up was done till the findings were normal.

### Statistical Analysis

The data were analyzed using IBM SPSS version 19.0. Categorical data were expressed as percentages (%). Continuous data were expressed mean (SD), if normally distributed, or median (IQR), if skewed. Characteristics between those who survived and those who died were compared by using either Chi-square test (categorical data) or Mann–Whitney U test (non-parametric data). We classified the children into three groups based on the types of treatment they had received: Steroid only, IVIg only or both. Characteristics between these groups were compared by using either Chi-square test (categorical data) or Kruskal Wallis test (non-parametric data). A univariate logistic regression analysis was done to identify risk factors associated with the primary outcome. A *p*-value of < 0.05 was considered as statistically significant.

## Results

A total of 134 MIS-C cases were included as per the WHO criteria. The median age of children was 84 months, and males constituted 66.7% of the cohort. The demographic, clinical and laboratory characteristics of children is described in Table 1.

**Table 1:** Demographic, clinical and laboratory characteristics of children with MIS-C.

**Parameters**	**Total number = 134**
**Demographic parameters**	
Male, *n* (%)	90 (66.7)
Age (months), median (IQR), range	84.0 (41.3, 132); 6–168
**Clinical parameters**	
Duration of illness (d), mean (SD)	5.8 (2.8)
Duration of hospital stay (d), median (IQR)	8.09 (6.0, 10)
Rash, *n* (%)	53 (39.6)
Gastrointestinal symptoms, *n* (%)	68 (50.7)
Respiratory symptoms, *n* (%)	53 (39.6)
Shock, *n* (%)	47 (28.9)
Non-purulent conjunctivitis, *n* (%)	95 (70.9)
Respiratory support, *n* (%)	84 (62.7)
Mechanical ventilation, *n* (%)	23 (17.2)
Co-morbidity, *n* (%)	32 (23.9)
**Laboratory parameters**	
RT-PCR or CBNAAT or IgG antibody to SARS-CoV-2, *n* (%)	134 (100)
Hemoglobin (g/dL), median (IQR)	10.1 (8.8, 11.3)
Total leucocyte count (TLC) (×10^5^/mm^3^), median (IQR)	13.7 (10.3, 18.5)
Neutrophils %, median (IQR)	85 (71, 90)
Lymphocytes %, median (IQR)	10 (6, 22)
Platelet count (×10^5^/mm^3^), median (IQR)	2.31 (1.3, 3.5)
ESR (mm/h), median (IQR)	50 (31.3, 75.8)
CRP (mg/dl), median (IQR)	66 (17.5, 106)
Procalcitonin (PCT) (ng/ml), median (IQR)	0.11 (0.1, 0.2)
IL6 (pg/ml), median (IQR)	11.94 (8.8, 32.5)
Serum ferritin (ng/ml), median (IQR)	363 (211, 734.2)
D-dimer (ng/ml), median (IQR)	3.63 (2.2, 9.1)
Serum fibrinogen (mg/dL), median (IQR)	198 (154, 280)
Serum triglycerides (mg/dL), median (IQR)	156 (112, 210)
SGOT (U/L), median (IQR)	58.5 (34.3, 104)
SGPT (U/L), median (IQR)	39.5 (25, 53.5)
Blood urea (mg/dL), median (IQR)	32 (23.9, 44)
Serum creatinine (mg/dL), median (IQR)	0.6 (0.4, 0.8)
Chest X-Ray Abnormal, *n* (%)	51 (38.6)
Myocardial dysfunction, *n* (%)	44 (33.3)

### Clinical Details

Fever was a universal finding (100%). Rash was seen in 40% cases (maculo-papular was most common, and 2 children had palpable purpura), and the median time of appearance from day of fever was 4 days. Joint symptoms was present in 12 cases (arthralgia = 10, and arthritis involving knee joint = 2). History of contact with confirmed COVID-19 cases (including household members) was present in 42 (31.3%) cases. In 15 children (8.35%), acute respiratory infection (ARI) in last 4 weeks was present. Thirty-two children (23.9%) had underlying co-morbidities involving respiratory (asthma = 6, interstitial lung disease [ILD] = 1), hematological (sickle cell disease = 5), neurological (seizure disorder = 4, storage disorder = 1, autoimmune encephalitis = 1), rheumatological (Systemic lupus erythematosus [SLE] = 3, Takayasu arteritis = 1, Systemic onset juvenile idiopathic arthritis [SOJIA] = 1), renal (nephrotic syndrome = 3, chronic kidney disease [CKD] = 2), cardio-vascular (rheumatic heart disease [RHD] = 1, patent ductus arteriosus (post-operative) = 1), and gastro-intestinal (chronic liver disease [CLD] = 1, Wilson's disease = 1) system. Gastro-intestinal (pain abdomen, vomiting and diarrhea), and respiratory symptoms were observed in 68 (50.7%) and 53 (39.6%) cases, respectively. Acute abdomen was seen in 6 of 68 (8.8%) children. Kawasaki disease (KD) features were present in 9 (6.7%) cases (typical KD = 1, atypical KD = 8).

### Laboratory Details

RT-PCR or CBNAAT or IgG antibody to SARS-CoV-2 was detected in all the cases. Laboratory details are provided in Table 1. Anemia was present in 90 (70.14%) cases (moderate to severe anemia in 66 [49.25%] cases). Leukocytosis was present in 66 (49.25%), and leucopenia in 6 (4.47%) cases. Neutrophilia was present in 104 (77.62%), and lymphopenia in 93 (69.4%) cases. Thrombocytosis was present in 37 (27.61%), and thrombocytopenia in 9 (6.72%) cases. Hypoalbuminemia was present in 85 (63.43%) cases. CRP level >100 mg/dl was seen in 96 (71.64%) cases. D-dimer was elevated in 58 (43.28%) cases. Ferritin level >500 ng/ml was seen in 51 (38%) cases. Elevation of amino-transferase enzymes >2 times upper limit of normal were seen as follows: AST only in 28 (20.89%), ALT only in 1 (0.74%), and both in 6 (4.47%) cases. Hyponatremia was present in 48 (35.82%) cases. SARS-CoV-2 IgG antibody titer of >50 U/ml was seen in 61 (45.52%) cases. Echocardiography evidence of myocardial dysfunction was present in 44 (33.33%) children. Troponin and Pro-BNP was elevated in 15 cases (both were done in selected cases only). Coronary artery abnormalities (CAAs) was seen in 14 (10.45%) cases (dilatation = 12, aneurysm = 2). Chest x ray was abnormal in 51 (38.63%) cases, and the abnormalities were as follows: cardiomegaly (*n* = 27; 20.15%), pulmonary edema (*n* = 13; 9.7%), and pneumonia (*n* = 12; 8.95%). Culture (blood or urine) was positive in 13 (9.7%) cases (Staphylococcus aureus = 6, Acinetobacter = 3, E. coli = 3, Klebsiella = 1).

### Hospital Course, Treatment and Outcome Details

Mean duration of illness was 5.8 days. Median duration of hospital stay was 8.09 days. Of 134 patients, 74 (55.22%) required admission to pediatric intensive care unit (PICU), and the median duration of PICU stay of was 4 days. Forty-seven children (35%) presented with shock (hypotension = 39), and 38 (81%) required support with vaso-active drugs. Shock was presented in 10 of 13 (76.9%) children with culture positive sepsis. Eight-four children (62.68%) required some form of respiratory support (face mask, nasal cannula, high-flow nasal cannula), of which 23 (27.38%) required mechanical ventilation. A total of 15 (11.2%) children died, and 2 got LAMA. The CAAs got normalized during follow-up in all but one child. Central nervous system (CNS) involvement was present in 12 (8.95%) study children during their hospital stay (encephalopathy = 7, features of meningitis = 2, stroke = 2, and lateral rectus palsy = 1). Two children developed acute kidney injury (AKI), one recovered and the other died. Twelve (8.95%) children did not require any immunomodulator therapy (IVIg or steroid), and in 122 (91.05%) cases, immunomodulator therapy was given (Steroid only = 88, IVIg only = 9, steroid *plus* IVIg = 25). Nineteen (14.18%) children received aspirin (3 to 5 mg/kg/day), and 17 (12.68%) received anticoagulation (low molecular weight heparin [LMWH]). All but one child received antibiotics. Among children who met the primary outcome, 9 (53%) had underlying co-morbidity: Asthma (*n* = 1), Wilson's disease (*n* = 1), sickle cell disease (*n* = 1), interstitial lung disease (*n* = 1), chronic kidney disease with severe hypertension (*n*= 1), SLE (*n* = 1), SOJIA (*n* = 1), storage disorder (*n* = 1), and nephrotic syndrome (*n* = 1).

We analyzed the characteristics of children who were discharged and who met the primary outcome (death) (Table 2). Male gender, underlying co-morbidity, hypotension at admission, coma at admission, thrombocytopenia, and elevated amino-transferase enzyme levels were significantly seen in children who met the primary outcome. We also compared the clinical and laboratory parameters among three sub-groups of immunomodulator therapy, and found lymphopenia to be significantly different among them (Table 3). We compared the outcomes in these three treatment sub-groups, and found no significant difference with respect to the primary outcome, and duration of PICU or hospital stay (Table 3). On logistic regression analysis, following factors were significantly associated with an increased risk of mortality: lymphopenia, thrombocytosis, hyponatremia, increased LDH (>300 U/L), and hypoalbuminemia (Table 4).

**Table 2:** Comparison of characteristics in the discharged and death groups.

		**Discharged** **(*n* = 117)**	**Died** **(*n* = 15)**	***P*-value**
Age (months), median (IQR)		84 (42, 120)	132 (41, 144)	0.15^a^
Gender, *n* (%)	Male	75 (64.1)	13 (86.7)	0.04^b^^*^
	Female	42 (35.9)	2 (13.3)	
Nutritional	Normal	103 (88.1)	15 (100)	0.31^b^
status, *n* (%)	Malnourished	4 (3.4)	0	
	Over-weight	10 (8.5)	0	
Co-morbidity, *n* (%)		23 (19.7)	7 (46.7)	0.02^b^^*^
Rash, *n* (%)		49 (41.9)	3 (20)	0.09^b^
Gastrointestinal involvement, *n* (%)		58 (49.6)	9 (60)	0.41^b^
Hypotension, *n* (%)		28 (23.9)	10 (66.7)	<0.001^b^^*^
Respiratory distress, *n* (%)		43 (36.8)	9 (60)	0.06^b^
Unconscious, *n* (%)		21 (17.9)	7 (46.7)	0.03^b^^*^
Myocardial dysfunction, *n* (%)		36 (30.8)	6 (40)	0.29^b^
Duration of fever /illness (d), median (IQR)		5 (5, 7)	5 (5, 7)	0.67^a^
Duration of hospital stay (d), median (IQR)		8 (6, 10)	7 (6, 10)	0.29^a^
Hemoglobin (g/dL) median (IQR)		10.1 (8.8, 11.3)	10.2 (9.3, 10.4)	0.97^a^
TLC (×10^3^/mm^3^), median (IQR)		13 (10.4, 18.3)	15.6 (8.8, 20)	0.46^a^
Lymphocyte, *n* (%)		10 (6, 23)	10 (9, 14)	0.58^a^
Platelet count (×10^5^/mm^3^), median (IQR)		2.38 (1.44, 3.5)	1.6 (0.9, 2.1)	0.007^a*^
ESR (mm/hr), median (IQR)		50 (30, 75)	58 (43, 100)	0.21^a^
CRP (mg/dL), median (IQR)		65 (16, 104)	70 (56, 122)	0.55^a^
Serum Ferritin (ng/ml), median (IQR)		346 (211, 693)	428 (267, 907)	0.66^a^
PCT (ng/ml), median (IQR)		0.1 (0.1, 0.2)	0.2 (0.1, 0.2)	0.27^a^
D-dimer (ng/ml), median (IQR)		3.6 (2.1, 8.5)	5.9 (3, 10.5)	0.13^a^
IL-6 (pg/ml), median (IQR)		12 (8.8, 34.5)	11.2 (10.1, 22.9)	0.9^a^
Sodium (meq/L), median (IQR)		138 (133, 140)	132 (130, 146)	0.42^a^
Albumin (g/dL), median (IQR)		3.1 (2.6, 3.7)	3.1 (2.7, 3.2)	0.69^a^
AST (U/L), median (IQR)		54 (33, 92)	104 (64, 131)	0.002^a^^*^
ALT (U/L), median (IQR)		36 (23, 49)	58 (44, 79)	0.001^a^^*^

a *Mann-Whitney U-test*.

b *Chi-Square test*.

* *P < 0.05*.

**Table 3:** Comparison of characteristics in the various treatment groups.

		**Steroids only (*n* = 88)**	**IVIg only** **(*n* = 9)**	**Steroids + IVIg (*n* = 25)**	***P*-value**
Age (months), median (IQR)		97 (48, 141)	41 (18, 72)	88 (36, 132)	0.13^a^
Gender, *n* (%)	Male	58 (65.91)	17 (68)	17 (68)	0.76^b^
	Female	30 (34.1)	8 (32)	8 (32)	
Nutritional status, *n* (%)	Normal	83 (94.3)	19 (76)	19 (76)	0.06^b^
	Malnourished	1 (1.1)	1 (4)	1 (4)	
	Overweight	4 (4.6)	5 (20)	5 (20)	
Co-morbidity, *n* (%)		22(25)	1(11.1)	6(24)	0.67^b^
Rash, *n* (%)		32 (36.4)	4 (44.4)	13 (52)	0.35^b^
Gastrointestinal involvement, *n* (%)		49 (55.7)	3 (33.3)	11 (44)	0.31^b^
Hypotension, *n* (%)		28 (31.8)	1 (11.1)	9 (36)	0.37^b^
Respiratory distress, *n* (%)		36 (40.9)	3 (33.3)	12 (48)	0.71^b^
Unconscious, *n* (%)		24 (27.3)	1 (11.1)	5 (20)	0.47^b^
Myocardial dysfunction, *n* (%)		31 (35.2)	2 (22.2)	9 (36)	0.72^b^
Duration of fever/illness (d), median (IQR)		5 (5, 6.5)	6 (5, 10)	5 (3, 7)	0.26^a^
Duration of hospital stay (d), median (IQR)		8 (6, 10)	9 (6, 12)	10 (7, 10)	0.18^a^
Duration of ICU stay (d), median (IQR)		2 (0–4)	0 (0–2)	3 (0–5)	0.28^a^
Death, *n* (%)		9 (10.2)	2 (22.2)	4 (16)	0.31^b^
Hemoglobin, median (IQR)		9.8 (8.5, 10.9)	9.6 (9.1, 11.6)	10.4 (9.5, 11.3)	0.36^a^
TLC (×10^3^/mm^3^), median (IQR)		13.7 (9.8, 17.8)	17.9 (12.4, 22.8)	13.7 (10.7, 21.5)	0.22^a^
Lymphocyte (%), median (IQR)		10 (5, 20)	30 (10, 36)	14 (6, 18)	0.046^a^^*^
Platelet count (×10^5^/mm^3^), median (IQR)		206 (134, 304.5)	387 (240, 512)	238 (118, 390)	0.11^a^
ESR (mm/hr), median (IQR)		50 (32.5, 77.5)	52 (36, 75)	65 (30, 78)	0.79^a^
CRP (mg/dL), median (IQR)		67 (24.5, 108)	98 (25, 106)	75 (13, 114)	0.76^a^
Serum Ferritin (ng/ml), median (IQR)		396 (208, 940)	510.9 (350, 1,000)	428 (284, 674)	0.74^a^
PCT (ng/ml), median (IQR)		0.12 (0.1, 0.3)	0.1 (0.1, 0.2)	0.1 (0.1, 0.2)	0.59^a^
D-dimer (ng/ml), median (IQR)		4 (2.3, 10.2)	3.63 (2.6, 6.4)	3.04 (2.2, 7.9)	0.63^a^
IL-6 (pg/ml), median (IQR)		13.74 (8.8, 30)	22.85 (11.2, 34.5)	11.05 (7.6, 34.5)	0.88^a^
Sodium (meq/L), median (IQR)		138 (132, 140)	135 (133, 138)	138 (133, 140)	0.82^a^
Albumin (g/dL), median (IQR)		3 (2.5, 3.5)	3.5 (3.1, 3.7)	3.2 (3, 3.6)	0.05^a^
Pro-BNP (pg/ml), median (IQR)		0 (0, 0)	0 (0, 0)	6,907 (1,991, 9,847)	-
Troponin (pg/ml), median (IQR)		206 (71, 4,767.8)	2,120 (2,120, 2,120)	40.05 (14.1, 66)	0.14^a^

a *Kruskal Wallis test*.

b *Chi-Square test*.

* *P < 0.05*.

**Table 4:** Univariate logistic regression analysis of factors association with primary outcome (death).

**Factors**		**Odds ratio (95% CI)**	***P*-value**
Age (<1 yr. as reference)	1–5	0.8 (0.1–8.6)	0.89
	>5	1.3 (0.15–11.6)	0.8
Anemia	No Anemia	Reference	
	Mild	4.1 (0.6–29.9)	0.16
	Moderate	0.9 (0.2–4.4)	0.92
	Severe	3.2 (0.7–14.1)	0.12
Leucocytosis		1.8 (0.1–6.8)	0.37
Leucopenia		2.1 (0.6–6.3)	0.23
Neutrophilia		-	0.47
Lymphopenia		1.2 (1.1–1.3)	0.04^*^
Thrombocytosis		7.1 (1.9–55.8)	0.03^*^
Thrombocytopenia		-	0.24
Hypofibrinogenemia		2.2 (0.7–6.6)	0.14
Increased CRP (>100 mg/L)		1.2 (0.4–3.4)	0.78
Increased D-Dimer		2.6 (0.7–8.9)	0.13
Hypernatremia		0.9 (0.3–2.6)	0.78
Hyponatremia		4.9 (2.2–34.8)	0.002^*^
Increased LDH (>300 U/L)		8.4 (1.7–40.9)	0.002^*^
Increased ALT (> 2 times of upper limit)		1.7 (0.6–5.3)	0.34
Increased AST (> 2 times of upper limit)		3.5 (0.6–19.9)	0.13
Low Albumin		4.5 (1.5–13.5)	0.01^*^
COVID-19 Ab Titer (>50 U/L)		1.7 (0.5–5.6)	0.39

* *P < 0.05*.

## Discussion

In present study, the median age of children with MIS-C was 7 years with males constituting 66.7% of the cohort. Fever was a universal finding. Rash was present in 40%, and conjunctivitis in 71% cases. Gastro-intestinal and respiratory symptoms were observed in 50.7% and 39.6% cases, respectively. Co-morbidity was present in 23.9% cases. Shock at admission was noted in 35%, and 27.38% required mechanical ventilation. Fifteen (11.2%) children died. The coronary abnormalities got normalized during follow-up in all except in one child. Initial choice of immunomodulation had no effect on the outcomes. Presence of underlying co-morbidity, lymphopenia, thrombocytosis, hyponatremia, increased LDH (>300 U/L), and hypoalbuminemia are the factors significantly associated an increased mortality.

In a recent systematic review analyzing the data of 953 cases from 68 article, the median age was 8 years with males accounting for 58.9% of the cases (12). In a largest single center study from India, the median age was 8 years with males constituting 61.29% of the cohort (13). In a multi-national study from Latin America (low-and middle-income country setting like India), the median age was 7 years with 54.7% being male (24). In our cohort, the median age of the children was 7 years in survivors compared to 11 years who died. In the above study from India, the corresponding figures were 8 years and 10.8 years, respectively (13). Majority of children who died were male (83.3%), which was higher than that (72.3%) reported in a systematic review (12). Fever was a universal finding (100%), which is in agreement with previous studies (6–8, 12–15). In our study, rash was reported in 40% cases compared to 57.14% in the previous Indian study (12), 68% in the Brazilian study (25), and 54.9% in a systemic review (12). Our study reported a higher rate of non-purulent conjunctivitis (71%) compared to 25.8% in the Indian study (13), 31.3% in the Turkish study (26), and 49.8% reported in the systematic review (12).

History of contact with confirmed Covid-19 cases (including household members) was present in 42 (31.3%) cases. This indicates that, contact history may not be important in a given case provided other criteria for MIS-C are met. In addition, treatment should not be delayed in severe cases pending confirmation of this history. Only 15 children (8.35%) had a documented history of acute respiratory infection in last 4 weeks. This is in agreement with the epidemiology of MIS-C, which indicates that majority of the cases develop 4–6 weeks after exposure to the virus (27). In our study, 23.9% had underlying co-morbidities, whereas it was 38.7% in the previous Indian study (13), 28.35% in Turkish study (26), and 15.8% in the Latin American study (24).

In our study, gastro-intestinal symptoms were observed in 50.37% cases, which was lower than that reported in other studies (13, 25, 26) but more than the Latin American study (24). The figure is still much lesser than that reported in the systematic review (85.6%) (12), which may be because of a larger sample size in the later. Acute abdomen was observed in 8.8% children in the present study compared to 9.5% in another study from Latin America (28). In contrast to the Latin American study, 2 of 6 children (33%) in the present study had acute appendicitis, and underwent surgery. Second most common were the respiratory symptoms (39.26%), which is similar to that reported in the Latin American study (24), but lower than that reported in the systematic review (12), the Brazilian study (25) and the previous Indian study (67.74%) (13). For unknown reason, the Turkish study reported a much lesser frequency of respiratory symptoms (7.4%) compared to all the studies (26).

In our study, evidence of myocardial dysfunction was present in 33.33% children, compared to 40.4% in the systematic review (12), 27% in the Brazilian study (24), 22.4% in Turkish study (25), and 44.83% in previous Indian study (13). CAAs were seen in 10.45% of our cases, which was similar to that reported in the systematic review (12). Lowest incidence of CAAs was reported in the Turkish study (6.45%) (26), and highest in the Brazilian study (27%) (25). The later figures may correspond to the incidence of Kawasaki disease (KD) in these countries, as KD and MIS-C probably share similar patho-physiologic mechanisms (29). The CAAs in our study got normalized during follow-up in all but one child, and this is in accordance with other studies (12, 13, 25, 26).

The prevalence of shock at admission was 35%, which is lower than the previously published studies (12, 13, 25). The shock may be because of myocardial dysfunction (secondary to micro-vascular damage, stress cardiomyopathy, and cytokine storm) or secondary bacterial infection or both (12, 25). The admission rate to PICU in our study was 55.2%, which was greater than the Turkish study (31.3%) (26), and lesser than that reported in the systematic review (73.3%) (12). The median duration of PICU stay was 4 days in our study, which is similar to all previous studies (the range being 4–8 days). Similar agreement was noted for duration of hospital stay (median was 8 days in our study as well as other studies). In our study, 27.38% children required mechanical ventilation, which is higher compare to other studies: 3% in Turkish study (26), 11% in Brazilian study (25), 22.5% in the previous Indian study (13), but lower than that reported (30%) in the systematic review (12). This indicates that children in our study were sicker compared to these studies. Most common indication of mechanical ventilation was shock (not respiratory failure).

Primary outcome (death) was common among children with underlying co-morbidity, which was in agreement with other studies. In the systematic review, older age, gastro-intestinal and cardio-vascular symptoms, less respiratory symptoms and absence of rash were associated with severe disease (12). In the Turkish study, presence of rash, bradycardia, hypoalbuminemia, and high neutrophil to lymphocyte ratio was noted in patients with severe disease (26). In the previous Indian study, presence of co-morbidity, absence of skin rash and a high serum ferritin level were significantly associated with mortality (13). In compared to these studies, male gender, underlying co-morbidity, hypotension & coma at admission, thrombocytopenia, and elevated amino-transferase enzyme levels were noted in children who met the primary outcome in our study. In the logistic regression analysis, lymphopenia, thrombocytosis, hyponatremia, increased LDH (>300 U/L), and hypoalbuminemia were the factors significantly associated with the occurrence of primary outcome (death or LAMA) in our study.

In our study, 8.95% cases did not require any immunomodulator therapy as they had mild disease with spontaneous recovery. Among the immunomodulator therapy, steroid alone was used in 72.13%, IVIg alone in 6.71%, and steroid *plus* IVIg in 20.5%. IVIg couldn't be administered in majority of the children due to financial constraints. However, there was no difference in the primary outcome (death), duration of PICU as well as hospital stay among these three treatment groups. This finding is vital for resource poor settings, where costlier treatments like IVIg are far from reach of the common man. Some cases of non-severe MIS-C have been reported to respond to oral prednisolone alone (30).

A higher rate of death (11.2%) noted in our study is similar to that reported in previous Indian study (12.9%) (13). Other Indian studies have reported mortality rate varying from 0 to 27.5% (17, 18, 31). However, these figures are much higher than those reported in the previous systematic review, and multi-center data from USA (12, 15). This may be because of a higher proportion of children with co-morbidity, delayed referral of admitted children, and delayed initiation of immunomodulator therapy in our study (13).

The present study has several strengths: (i) multi-center study with large sample size, (ii) data reported from LMIC (low- and -middle income country) setting, (iv) reported wide array of clinical, laboratory, and serological data pertinent to MIS-C, and (iv) analyzed treatment response in a large cohort of MIS-C. However, the study has certain limitations. Being tertiary care referral hospitals, the overall spectrum of MIS-C might not have been reflected, so the results may not be generalizable. A lesser number of children received IVIg only; this may not truly reflect the scenario in which these children should have been managed (as per ACR guideline). As the data were collected retrospectively, the study has its inherent limitations of this type of observational study.

## Conclusions

MIS-C can have myriad of manifestations. Presence of underlying co-morbidity, lymphopenia, thrombocytosis, hyponatremia, increased LDH (>300 U/L), and hypoalbuminemia are the factors associated with an increased mortality. No difference in outcome was noted with steroid or IVIg. Coronary artery abnormalities resolve in nearly all cases.

## Data Availability Statement

The original contributions presented in the study are included in the article/Supplementary Material, further inquiries can be directed to the corresponding authors.

## Ethics Statement

The studies involving human participants were reviewed and approved by Institute Ethics Committees of five participating sites: SCB Medical College, Cuttack; AIIMS, Bhubaneswar; VIMSAR, Burla; IMS & SUM Hospital, Bhubaneswar; and MKCG Medical College, Berhampur. Written informed consent to participate in this study was provided by the participants' legal guardian/next of kin.

## Author Contributions

SnN, PP, BB, SA, and RD: concept, abstracting data from papers, and managing data. SaN, RD, and AS: performing statistical analysis, interpreting data, and making statistical inferences. SnN, RD, PJ, and PP: writing first draft of the paper. DR, AM, DP, KG, and SM: abstracting data from papers, making inferences, and critical review of the paper. All authors have approved the version to be published. RD and DR will jointly act as guarantors.

## Members of the Eicomisc Study Group

Subash Chandra Kumar Majhi, VIMSAR, Burla, Sambalpur; Sitanshu Meher, VIMSAR, Burla, Sambalpur; Chandrakant Poddar, VIMSAR, Burla, Sambalpur; Jyotiprakash Sahoo, SCB Medical College, Cuttack; Martina Mohanty, SCB Medical College, Cuttack; Satya Brata Padhy, VIMSAR, Burla, Sambalpur; Bijan Kumar Nayak, VIMSAR, Burla, Sambalpur; Shovendra Kumar Dash, VIMSAR, Burla, Sambalpur; Soumya Ranjan Meher, VIMSAR, Burla, Sambalpur; Pranab Kumar Panigrahi, VIMSAR, Burla, Sambalpur; and Srikanth, Asam, IMS & Sum Hospital, Bhubaneswar.

## Conflict of Interest

The authors declare that the research was conducted in the absence of any commercial or financial relationships that could be construed as a potential conflict of interest.

## Publisher's Note

All claims expressed in this article are solely those of the authors and do not necessarily represent those of their affiliated organizations, or those of the publisher, the editors and the reviewers. Any product that may be evaluated in this article, or claim that may be made by its manufacturer, is not guaranteed or endorsed by the publisher.

## Supplementary Material

Supplementary Figure 1 Schematic approach to children managed in the collaborating centers.
