# Supplementary figures and images for: Eastern India Collaboration on Multisystem Inflammatory Syndrome in Children (EICOMISC): A Multicenter Observational Study of 134 Cases
Source: Front Pediatr. 2022 Mar 11;10:834039. doi: 10.3389/fped.2022.834039 (PMC8963178; doi:10.3389/fped.2022.834039)

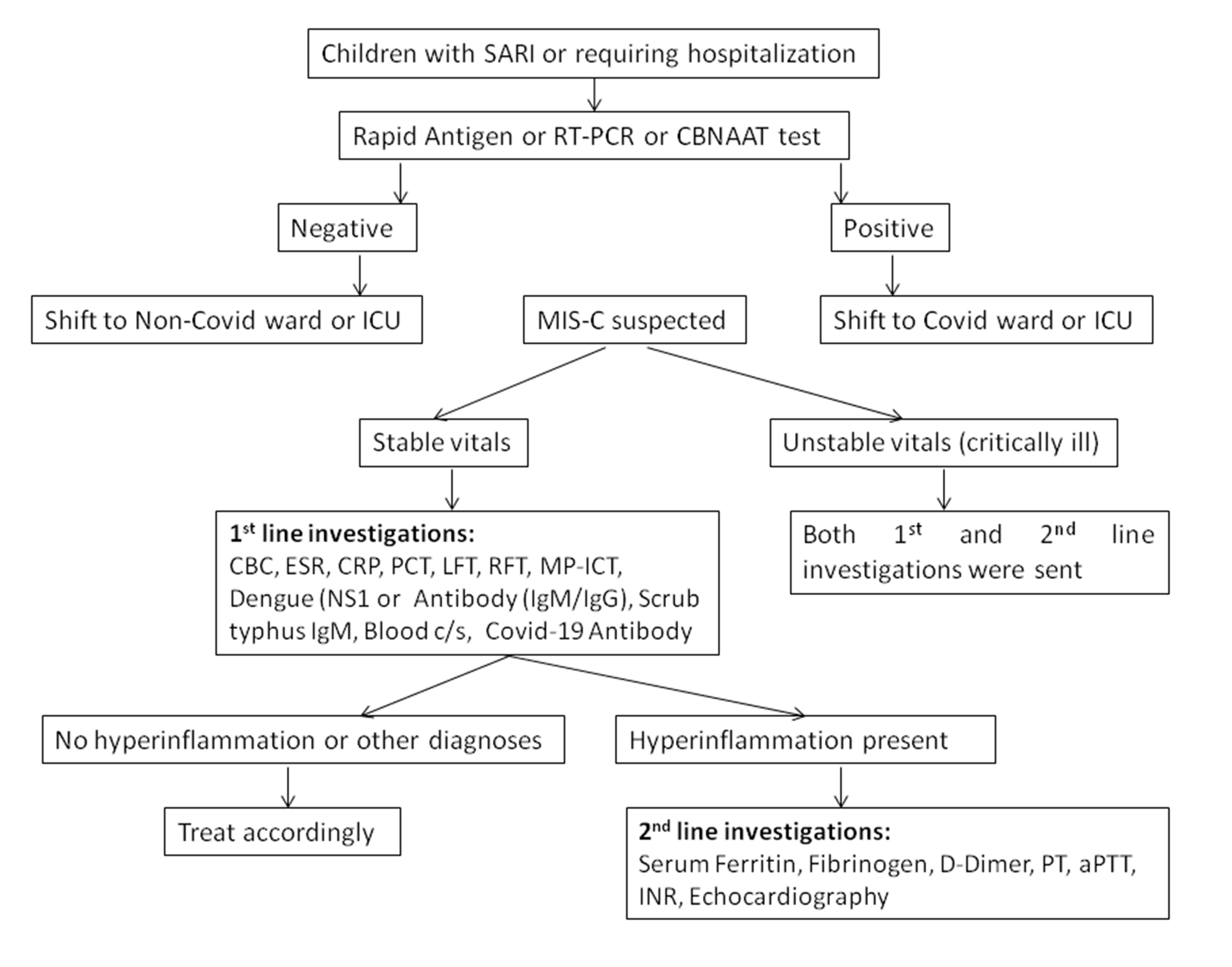

Supplement: Supplementary Figure 1 — Schematic approach to children managed in the collaborating centers. [file Image_1.tif]
